# Effect of sandblasting and MDP based primer application on the surface topography and shear bond strength of zirconia: An *in vitro* study

**DOI:** 10.12688/f1000research.166325.1

**Published:** 2025-07-02

**Authors:** Vaishnavi Jalaj, Vignesh Kamath, Umesh Pai, Srikant Natarajan, Shanmethaa S, Akshita Gupta

**Affiliations:** 1 Manipal College of Dental Sciences Mangalore, Manipal Academy of Higher Education, Manipal, Karnataka, 576104, India; 2 Department of Prosthodontics and Crown and Bridge, Manipal College of Dental Sciences Mangalore, Manipal Academy of Higher Education, Manipal, Karnataka, 576104, India; 3 Department of Oral Pathology and Microbiology, Manipal College of Dental Sciences Mangalore, Manipal Academy of Higher Education, Manipal, Karnataka, 576104, India

**Keywords:** Bond strength, Primer, Sandblasting, Surface treatment, Zirconia, 10-MDP

## Abstract

**Background:**

Recently, prosthodontic utilization of zirconia-based materials has increased. There is insufficient research on the impact of MDP primers on zirconia bonding. This research was conceptualized to understand the impact of various surface preparation techniques, utilizing different abrasive powders and MDP primers, on the adhesive strength offered by zirconia copings.

**Methods:**

Discs composed of 3Y-TZP zirconia measuring 18 mm in diameter and 4 mm in thickness were fabricated by milling fully sintered zirconia blanks that had been isostatically pressed. The samples were then classified into three categories. Group A, MDP primer; Group B, sandblasting with aluminum oxide; Group C, MDP priming and sandblasting with aluminum oxide. Scanning electron microscopy, atomic force microscopy, shear bond strength tests, and mode of failure analyses were performed.

**Results:**

Thirty samples were analyzed. The shear bond strength test results showed that Group A had an average score of 8.62 ± 3.18 Group B had a lower value of 5.50 ± 1.78, and Group C had the highest value of 16.05 ± 8.91. The mode of failure among the three groups was found to have no statistically significant changes.

**Conclusion:**

The application of MDP primers after air abrasion markedly enhanced the shear bond strength and surface roughness of zirconia, as opposed to the effects of each isolated method.

## 1. Introduction

The use of zirconia in clinical dentistry has increased in recent decades, owing to its aesthetic value, biocompatibility, and superior mechanical properties. Zirconia exhibits increased fractural toughness and high flexural strength, and harmonizes translucency and opalescence analogous to natural teeth, making it the foremost choice for replacement of teeth. ^1^ With a flexural strength of approximately 900–1200 MPa, 9–10 MPa of fracture resistance and a compression resistance of 2000 MPa, zirconia is among the most sought-after restorative treatments. ^2^ Zirconia is primarily a polycrystalline ceramic that lacks the glass phase. It has three different crystalline structures, monoclinic, cubic, and tetragonal, which vary based on pressure and temperature. The variation in these crystalline structures imparts distinct optical and mechanical attributes to each type, making them versatile for clinical use. ^3^ The zirconia commonly used in dentistry are tetragonal zirconia polycrystals, which possess the greatest fracture resistance and flexural strength. ^4^ The addition of 3 mol% yttrium oxide in the form of a stabilizer to the tetragonal phase, resulting in the formation of yttria-stabilized tetragonal zirconia polycrystal (3Y-TZP), is the form of zirconia used primarily in dentistry. Various compositional modifications have been made to zirconia by increasing its yttria content to enhance its properties. ^5^ Despite the advancements in improving the clinical adaptability of zirconia, it presents considerable challenges in being retained for longer durations and adhering to resin cements. Zirconia exhibits chemical inertness owing to its inability to be activated by etching with hydrofluoric acid. ^6^ This also hinders adhesion because of the lack of silanization with silica-based primers. This hindrance in bonding predisposes zirconia to clinical failure, the risk of caries, and discomfort to the patient. ^7^ Furthermore, intraoral contamination of zirconia with blood, saliva, and other contaminants can alter its adhesion to resin cements.

Although conventional methods of cementation can be utilized for zirconia, extensive surface modifications that enable greater marginal seal and fracture resistance are favorable for a more durable restoration. Various surface modification techniques and methods have been studied to improve adhesion between zirconia, resin cement, and tooth surfaces. Methods such as Airborne-Particle Abrasion (APA), ^8^ Tribochemical Silica Coating (TBC), ^9^ Plasma Treatment, ^10^ high-concentration acid etching, ^11^ and methacryloyloxydecyl dihydrogen phosphate (10-MDP) ^12^ have been investigated for their beneficial outcomes.

One of the most effective methods for generating micromechanical adhesion in zirconia restorations is airborne-particle abrasion, along with the use of aluminum oxide particles coated with silica. The insertion of aluminum particles covered with silica altered the surface, resulting in an abrasive effect. Increased surface reactivity for silanization and enhanced contact with resin cement have been observed. ^13^ Nevertheless, the enhanced ability to be retained following airborne-particle abrasion is linked to the development of small cracks in the zirconia material, which could negatively impact the long-term efficacy of repair. ^14^

In addition to mechanical approaches, encouraging outcomes have been demonstrated when chemical agents are used to facilitate the bonding of resin cement with zirconia. ^15^ The application of 10-methacryloyloxydecyl dihydrogen phosphate (MDP) treatment, a dentin primer, or a silane agent consisting of an ester of phosphate, has been frequently used to chemically attach zirconia to resin cement. By incorporating a divalent phosphoric end into the MDP monomer, a chemical link was established with the zirconium surface, leading to an increase in the durability of the bond. ^16^ Although there has been a focus on bonding, the combined effect of these surface modifications on the surface topography of zirconia and its influence on bond strength have seldom been studied using high-resolution imaging. ^16^ Employing varying particle sizes, pressures, times, and primers across studies constrains their clinical adaptability. In view of these concerns, this investigation was conceptualized to determine the synergistic influence of sandblasting and MDP-based primers on the shear bond strength and surface topography of zirconia using high-resolution imaging techniques such as SEM. This extensive analysis of both the micromechanical and chemical processes involved in the bonding of zirconia provides a novel perspective for proposing a uniform surface treatment procedure for zirconia restorations. Hence, this study aimed to determine the effects of sandblasting and MDP-based primers on the shear bond strength and surface topography of zirconia.

## 2. Methods

### 2.1 Study design and study setting

This in vitro experimental study was carried out to examine the effect of sandblasting and MDP primers on the surface topography and shear bond strength of zirconia. The study was initiated after obtaining ethical approval from the Institutional Ethics Committee (Protocol Ref No: 23119, obtained on 15 January, 2024) and was carried out in the Prosthodontics and Crown and Bridge Department of the Manipal College of Dental Sciences, Mangalore.

### 2.2 Sample size and sampling

Sample size estimation was conducted according to data provided in a previously published study on similar subjects. ^17^ The power of the study was assumed to be 80%, and the significance level was set at 5%. The corresponding Z scores were 0.8416 and 2.3939, respectively. A standard deviation of 0.33 MPa and a clinically relevant minimum difference of 0.5 MPa were considered to ensure 10 samples per group. To obtain a clinically significant difference (d) of 0.5, a standard deviation of 0.33 was considered. The required sample size for each group was 10. Given that each of the three experimental groups included ten samples, the overall sample size was 30.

### 2.3 Methodology

**2.3.1 Sample preparation**

In this study, zirconia discs were fabricated by milling fully sintered zirconia blanks isostatically pressed with zirconia blanks. Thirty discs of 3 mol% yttria-stabilized tetragonal zirconia polycrystal (3Y-TZP zirconia) measuring 18-mm in diameter and 4 mm thick were fabricated. The discs were categorized into three groups of 10 each based on the surface treatment received as follows:

Group A: surface was treated with MDP primer

Group B: aluminum oxide was used to sandblast the surface of the discs

Group C: sandblasted with aluminum oxide and surface treated with MDP primer

Sandblasting was performed with alumina particles of 110 μm size, under 50 psi pressure with zirconia discs at 10 mm from the nozzle for a duration of 5 min, moving the nozzle in a uniform direction. Following surface treatment, before the application of the MDP primer, the zirconia discs were rinsed in an ultrasonic cleaner using distilled water for 10 min.

**2.3.2 Surface characterization**

The surface topography of the zirconia discs included two discs that were randomly selected from each of the experimental groups and then assessed using Atomic Force Microscopy (AFM). Further, structural analysis of two independent discs chosen at random from each group was performed using scanning electron microscopy (SEM). To evaluate the chemical structure of the discs subjected to various surface treatments, energy-dispersive X-ray analysis was conducted using SEM.

*Surface Microstructure Analysis using Scanning Electron Microscopy (SEM)*

For microstructure analysis, two discs from each experimental group were chosen at random. Before the analysis, the samples were sputtered with a 10 nm gold layer to improve conductivity. Subsequently, a Zeiss EVO MA 18 scanning electron microscope (Carl Zeiss, Jena, Germany) was used for the surface analysis. To explore the composition of zirconia discs at the elemental level after the three surface modifications, Scanning Electron Microscopy (SEM) in conjunction with energy dispersive X-ray analysis (EDAX, Oxford EDS (X-act), Abingdon, United Kingdom) was performed.

*Surface Topography Analysis with Atomic Force Microscopy (AFM)*

The surface roughness was analyzed on two randomly chosen discs from each group via Atomic Force Microscopy (AFM, Innova SPM AFM, Bruker, Massachusetts, USA) operated in non-contact operational mode. An AFM cantilever fitted with magnetoresistive sensors at the tip was used for the imaging. Assessments were performed at three randomly selected sites per disc, with a reference spot dimension of 50 × 50 μm.

**2.3.3 Preparation of samples for estimating the Shear Bond Strength**

The surface-treated discs were coated with a silane coupling agent (Silano, Angelus, Londrina, Brazil) using a microbrush and air-dried. Thereafter, two layers of the adhesive (Single Bond Universal Adhesive, 3M ESPE, St. Paul, Minnesota, USA) were coated, agitated gently, and the solvent was evaporated by an air stream. According to the manufacturer’s instructions, the adhesive was cured for 10 s. Owing to the restricted dimensions of the disc, a maximum of five samples were inserted per disc making use of 6 discs to produce a combined total of 30 samples. Five Tygon tubes, each with an inner diameter and a height of 5 mm, were used to cut the discs. Composite resin cement (Fusion Ultra D/C) was meticulously mixed and dispensed into Tygon tubes and placed firmly against the surface of the disc. Following the manufacturer’s instructions, the samples were subjected to light-curing for 40 s. The additional four Tygon tubes were linked to the zirconia discs. After polymerization, Tygon tubes were extracted from the discs using a precision blade. The extracted specimens were stored at room temperature for 24 h prior to testing to facilitate additional plasticization.

**2.3.4 Shear bond strength test**

**Figure 1.**
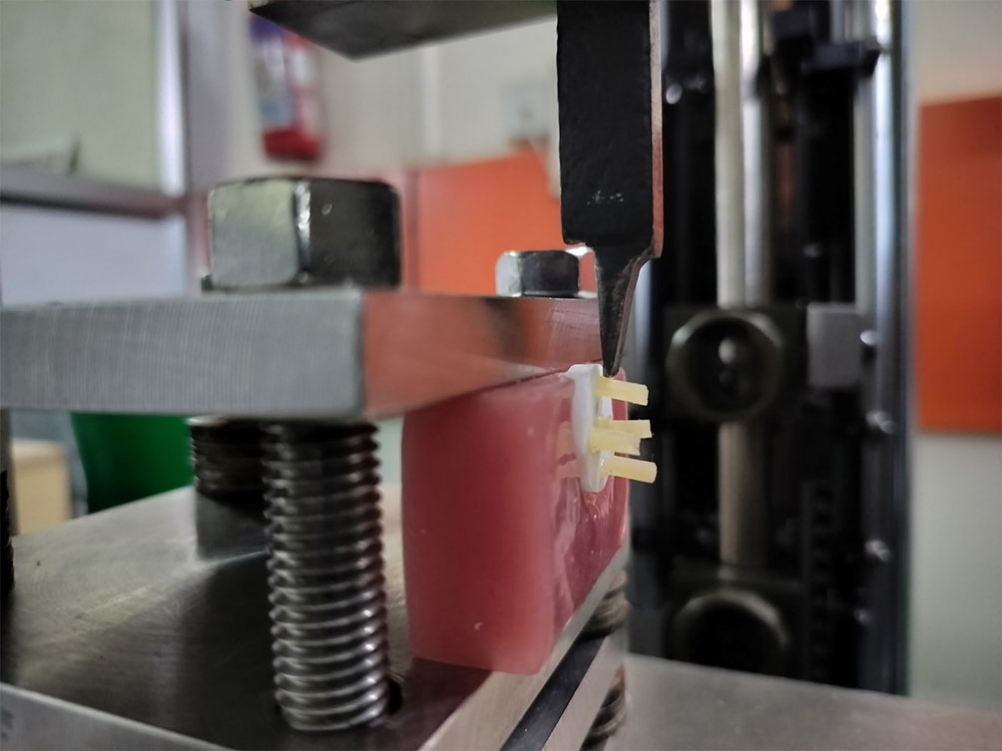
Shear bond strength testing of zirconia disc using universal testing machine.

For the shear bond strength analysis, a universal testing machine (Zwick/Roell Z020, Ulm, Germany) was used ( Figure 1). The modified blade was custom-made and manufactured at the Hebich Technical Training Institute (HTTI) in Mangalore, Karnataka, India. The blade was placed perpendicular to the resin and ceramic interface. The zirconia discs were positioned perpendicular to the blade with the aid of a heat-cured PMMA block of dimensions 40 mm × 20 mm × 25 mm to ensure stability and prevent mechanical displacement throughout the testing. A shear pressure of 0.5 mm/min was maintained at a constant crosshead rate until the test piece fractured. The shear bond strength (megapascals-MPa), the greatest failure load (Newtons-N) in the numerator, and the dimension of the adhesive area (in mm ^2^) in the denominator were determined.

$$\text{Shear bond strength}\;\left(\mathrm{MPa}\right)=\frac{\text{Failure load}\;\left(N\right)}{\text{Surface area}\;\left({\mathrm{mm}}^{2}\right)}$$ (1)

**2.3.5 Mode of failure analysis**

The modes of failure were classified as cohesive, adhesive, and mixed, using a compound zoom microscope (Olympus, Olympus Scientific Solutions America Corp., Waltham, USA). The magnification was set at 40×. Two different samples drawn from each experimental group were investigated using SEM to assess the surface microstructure.

### 2.4 Data analysis

Statistical analysis was performed using IBM SPSS (Statistical Package for Social Sciences) version 26. For the shear bond strength values obtained from the three groups (A, B, and C), normality tests were used. One-way analysis of variance (ANOVA) was performed to compare shear bond strength values between the groups. Tukey’s Honestly Significant Difference (HSD) post-hoc test was performed to establish a significant difference between the groups. Furthermore, a chi-square test was conducted to evaluate the association between the mode of failure and the experimental groups. For all statistical analyses, the p value was kept at < 0.05 to imply statistical significance.

All assessment tools used in this study were utilized following the manufacturer’s instruction of Standard operating protocol. They were used with licensed institutional access and did not require additional copyright license for use.

## 3. Results

This study evaluated the effects of sandblasting and MDP primers on the shear bond strength, surface microstructure, topography, and elemental composition of zirconia. Following surface modifications, the 30 zirconia discs were analyzed using Scanning Electron Microscopy (SEM) along with Energy Dispersive X-ray Spectroscopy (EDS) for microstructure analysis, and the surface topography was assessed using Atomic Force Microscopy.

### 3.1 Surface topography

The surface topography was determined using Atomic Force Microscopy (AFM) ( Table 1). Group C had the greatest surface roughness, which was suggestive of the highest surface modification. Group A exhibited the lowest level of roughness, indicating the lowest surface change.

**Table 1.** Surface topography of the zirconia discs assessed with Atomic Force Microscopy.

Groups	20 nm		50 nm	
Group A	Rq	17.6 nm	Rq	27.4 nm
	Ra	13.1 nm	Ra	19.8 nm
	Rmax	171 nm	Rmax	292 nm
Group B	Rq	57.1 nm	Rq	10.2 nm
	Ra	44.9 nm	Ra	75.8 nm
	Rmax	587 nm	Rmax	1729 nm
Group C	Rq	86.6 nm	Rq	170 nm
	Ra	67.3 nm	Ra	125 nm
	Rmax	663 nm	Rmax	1790 nm

Rq - Root Mean Square Roughness; Ra - Average Roughness; Rmax - Maximum Roughness Depth.

### 3.2 Surface microstructure analysis

**Figure 2.**
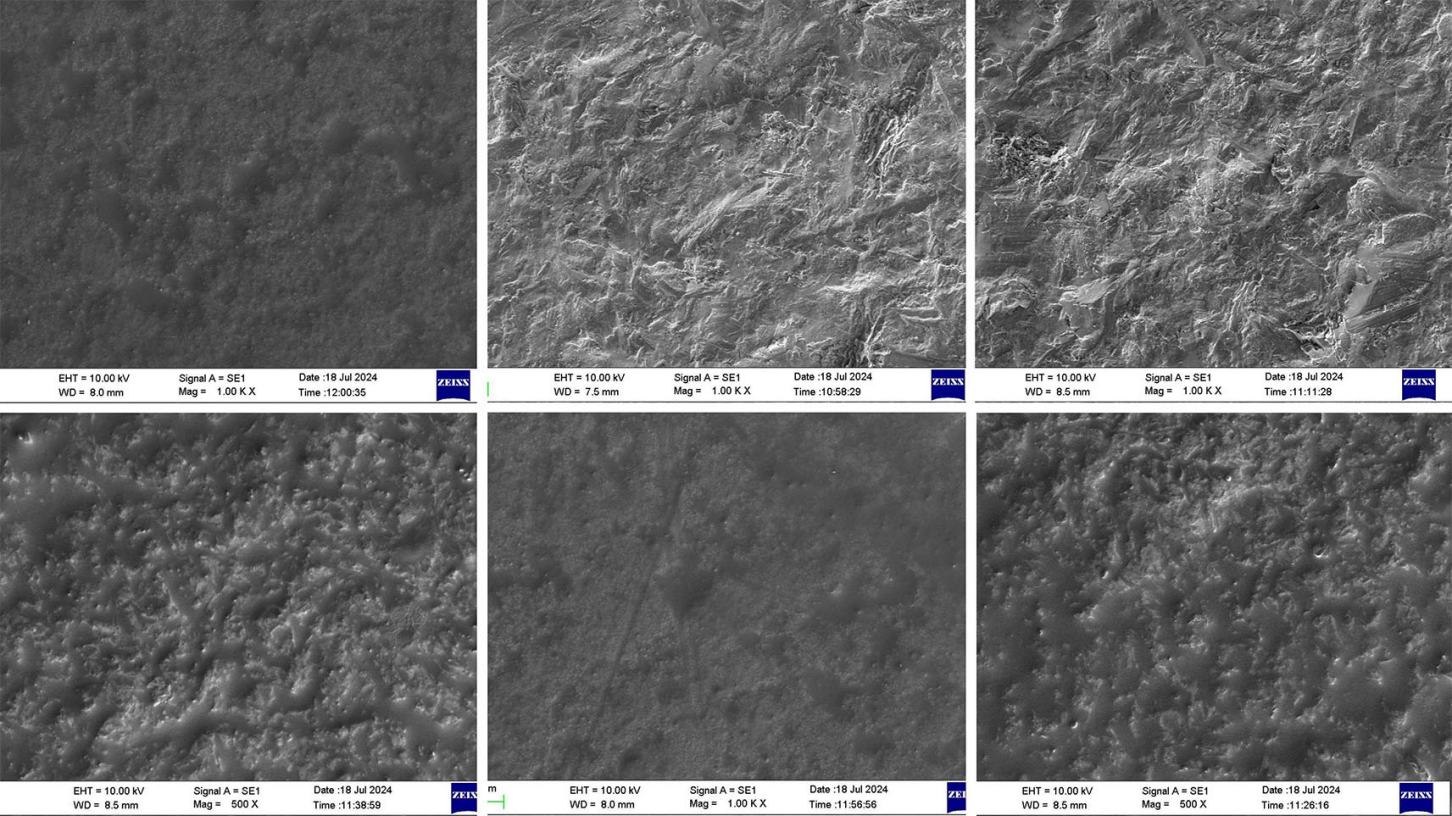
Scanning Electron Microscopy (SEM) image of the zirconia discs after being subject to surface modifications. Group A - left: surface was treated with MDP primer. Group B - middle: surface treated with aluminum oxide sandblasting. Group C - right: surface treated with aluminum oxide sandblasting and treated with MDP primer. The upper row depicts surfaces at 1000× magnification, and the lower row depicts surfaces at 500× or 1000× magnification.

SEM analysis was performed to examine the microstructure of the zirconia discs after the different surface modifications ( Figure 2). Group A, treated with the MDP primer, showed minimal irregularities with an almost smooth surface, suggestive of a less favorable surface for micromechanical retention. Group B, in which aluminum oxide was used for sandblasting, showed a very smooth surface analogous to a coating, indicating a poor surface for micromechanical retention with low bond strength. The surfaces of the discs in group C were treated with MDP primers and sandblasted with aluminum oxide. This group exhibited a rough surface with irregularities comparable to etching, indicative of good micromechanical retention owing to the formation of ridges and grooves seen over the surface.

The **elemental composition** of the surface-treated discs were examined using energy dispersive X-ray spectroscopy ( Table 2). The analysis determined in all the groups detected the elements zirconium (Zr), aluminum (Al), oxygen (O) and hafnium (Hf ). The elements were found in varying concentrations owing to differences in the surface treatments used. Of the three experimental groups, group A showed the highest zirconium (65.37 wt%), while group B had the highest aluminum (6.08 wt%). Group C had the lowest zirconium (55.45 wt%). It can be inferred from these findings that group A had the least surface modification, whereas group C had the most surface modification.

**Table 2.** Elemental composition of the zirconia discs assessed through SEM analysis.

Group	Zr (wt%)	O (wt%)	Al (wt%)	Hf (wt%)
A	65.37	32.38	0.42	1.83
B	60.19	32.08	6.08	1.65
C	55.45	36.41	5.17	2.98

Expressed as weight percentage for all categories. Zr - Zirconia; O - Oxygen; Al - Aluminum; Hf - Hafnium.

### 3.3 Shear bond strength

The shear bond strength of the experimental groups was assessed using a one-way analysis of variance (ANOVA) ( Table 3). Group A had a mean shear bond strength of 8.62 ± 3.18 MPa; Group B had a strength of 5.50 ± 1.78 MPa; and Group C had the highest strength at 16.05 ± 8.91 MPa. One-way ANOVA showed a statistically significant difference among the tested groups (p = 0.003), suggesting a difference in mean shear bond strength in relation to the groups. A post-hoc test was used to assess which of the specific groups showed a difference. The difference between Groups B and C was statistically significant (p = 0.007), whereas there was no significant variation with respect to the other groups. This indicates that the shear bond strength in group C was significantly greater than that in group B. Clearly, the use of sandblasting combined with the MDP primer improved the shear bond strength.

**Table 3.** Shear bond strength comparison using One-way ANOVA & Post Hoc Tukey Test.

Group	Mean ± SD	P value	Group comparison	Test statistic	Posthoc significance
Group A	8.62 ± 3.18	0.003	Group A-Group B	3.4	0.688
Group B	5.50 ± 1.78		Group B-Group C	-8.6	0.007
Group C	16.05 ± 8.91		Group A-Group C	-5.2	0.198

p < 0.05 considered statistically significant.

### 3.4 Mode of failure

Failure modes were analyzed after assessing the shear bond strength, and the modes in all three groups were categorized as adhesive, cohesive, and mixed ( Table 4). Groups A and B showed adhesive failure, whereas Group C presented cohesive failure. Analysis of the failure modes showed that 80% of the instances in Group A exhibited adhesive failure, whereas 20% displayed mixed failure. Conversely, all patients in Group B exhibited adhesive failure. Group C showed a varied distribution characterized by 40% cohesive, 40% mixed, and a mere 20% adhesive. However, there was no statistical significance (p = 0.5089) in the distribution of failure modes with respect to the three groups.

**Table 4.** Mode of failure analysis.

	Group A	Group B	Group C	Chi Square	P value
Adhesive	4(80)	5(100)	1(20)	3.3	0.5089
Mixed	1(20)	0(0)	2(40)		
Cohesive	0(0)	0(0)	2(40)		

Values expressed as N (%) – number of samples (percentage within the group).

Chi square test applied: p < 0.05 considered statistically significant.

## 4. Discussion

The findings from this study suggest that surface modification of zirconia improves the shear bond strength between resin cements and zirconia. In particular, the integrated use of aluminum oxide air abrasion along with the 10-methacryloyloxydecyl dihydrogen phosphate (MDP) primer for zirconia resulted in a remarkable improvement in the bond strength.

### 4.1 Surface topography and roughness

The data obtained from Atomic Force Microscopy indicated the highest surface roughness in group C, which underwent combined surface modification by sandblasting and MDP primer application. Substantial surface roughness was noted in group B, which was subjected to sandblasting alone, and the lowest surface roughness was observed in group A, with only MDP primer application. Studies have shown alterations in the surface roughness of zirconia with larger-sized alumina particles due to changes in surface topography. The formation of irregular grooves, microcracks, and abraded texture is produced by sandblasting. ^18^ Microporosities are formed on the surface of zirconia as a result of sandblasting, which contribute to improved resin bonding. ^19^

The elemental composition of the zirconia discs was assessed using energy-dispersive X-ray spectroscopy. It revealed the highest zirconium content in group A, while group B had moderate aluminum content, and group C showed the highest aluminum and oxygen retention after surface modification. These findings in group C are similar to those of earlier studies that suggested phosphate-zirconia bond formation due to chelation in relation to zirconium ions as a result of the chemical bond with the MDP primer. ^20, 21^ The reduction in zirconium content accompanied by increased oxygen and hafnium content of group C can be attributed to the process of surface oxidation.

### 4.2 Shear bond strength

**Figure 3.**
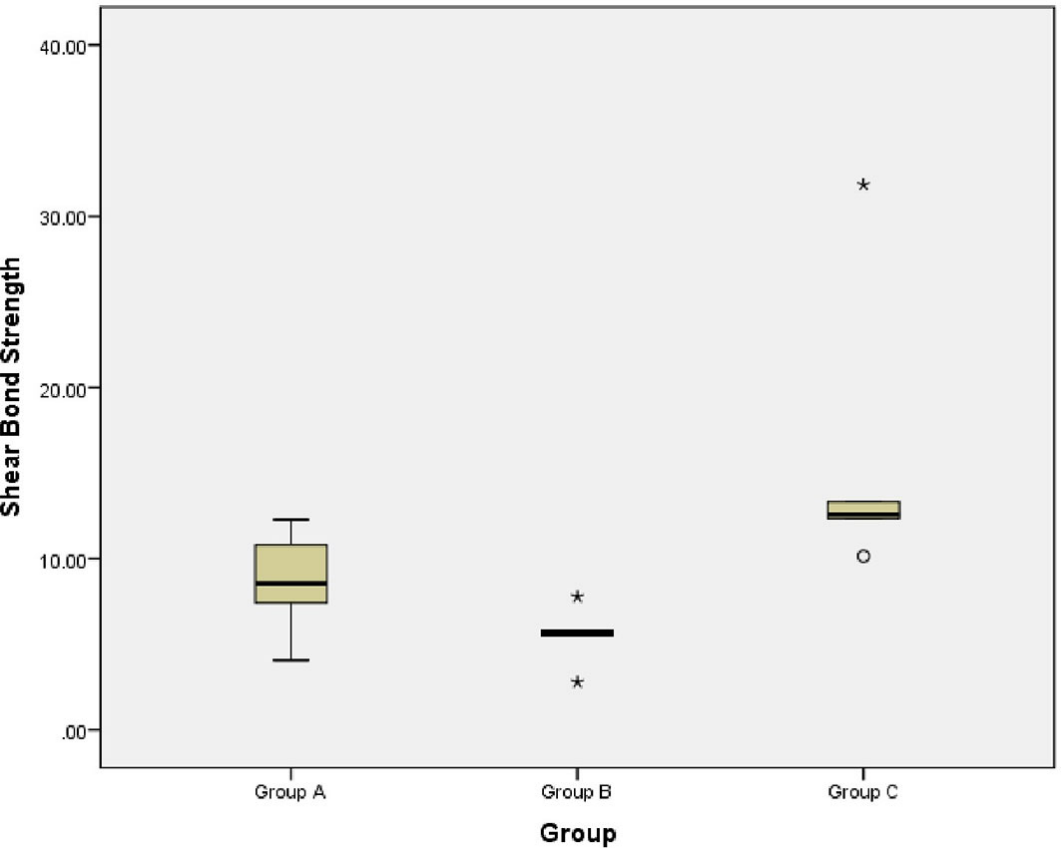
Box plot depicting shear bond strength (MPa) of zirconia discs after three surface treatments. One-way ANOVA was performed to compare the group means, p < 0.05 considered statistically significant.

The findings revealed that group C, which was subjected to both aluminum oxide sandblasting and MDP primer application, exhibited the highest shear bond strength among the three experimental groups ( Figure 3). Group A, which received surface treatment with the MDP primer, had the lowest bond strength, while group B, which was treated with aluminum oxide sandblasting, had moderate bond strength. In the post hoc test, group B as well as group C exhibited significant differences (p = 0.007), suggesting a higher shear bond strength in group C than in group B. These results are in agreement with previous research that had similar findings, wherein combined surface treatment with MDP primer and sandblasting profoundly increased bonding to zirconia. ^22^ This increase in bond strength can be ascribed to the enhanced surface roughness following sandblasting with aluminum oxide and improved micromechanical retention. ^23^ The MDP primer, owing to the presence of phosphate monomers, results in strong and direct chemical bonds between the resin cements and zirconia. ^24^ Another study by Xiong et al. reported that airborne particle abrasion along with an MDP primer proved to have the highest adhesive strength as opposed to the use of a singular surface modification. ^25^ Alumina-particle air abrasion functions by stimulating the zirconia surface, augmenting its wetting ability, producing microscale mechanical bonding, and removing contaminants of organic or inorganic compounds from the zirconia surface. Furthermore, air abrasion induces a phase transformation from tetragonal to monoclinic, which imparts fracture resistance and enhances the biaxial flexural strength of zirconia. ^26^ MDP primers play a favorable role by limiting the hydrolytic degradation of zirconia and strengthening bond stability over a long period of time. ^27^ The synergistic effect produced when these surface modifications are utilized increases the shear bond strength of zirconia.

### 4.3 Mode of failure

The analysis of the mode of failure provides an overview of the bond strength. Evidence on the mode of failure has previously shown that the presence of cohesive or mixed modes of failure indicates strong bonds, whereas adhesive failure is suggestive of weak bonding between the resin and zirconia. ^28^ In this study, a proportional distribution of both mixed and cohesive failures was present in group C, implying that bond strength was stronger and more durable in zirconia treated with sandblasting and MDP primers. Previous studies have shown that surface treatment with sandblasting and MDP primers results in cohesive or mixed failures, suggesting greater adhesion by this method. ^29^ Although no statistically significant difference was noted (p = 0.5089), the distribution of mixed and cohesive failures in group C was critical in interpreting the results in the context of other findings. It is likely that the small sample size may have hindered the statistical significance of the results. Consistent with the current investigation, the literature has established that durable bonding with respect to resin cement and zirconia can be achieved by employing MDP-based procedures. ^16, 30^

Currently, the recommended approach is to use alumina airborne-particle abrasion in combination with primers that include a 10-MDP primer. ^18^ Evidence from this study substantiates the superiority offered by the synergistic effect of alumina airborne-particle abrasion along with the 10-MDP primer in enhancing and sustaining the bond strength of resin cement and zirconia. Contrasting findings have also been established on the use of aluminum oxide air abrasion particles, wherein varying particle sizes have been shown to cause failure of zirconia crowns. ^31^ Alumina air abrasion has been shown to result in sharp surface defects that can potentially act as stress concentration points that predispose Zr to failure. ^32^ This could likely threaten the durability of the strong bonding in relation to resin cement and zirconia. Clearly, clinical decision-making should be preceded by evidence-based selection of surface treatment protocols for zirconium. Alumina air abrasion should be performed considering the size of the particles and the pressure to be used to achieve optimal bonding and structural integrity of zirconia crowns.

Despite the elaborate findings of this study, their interpretation should be performed considering the various limitations. The relatively small sample size could have affected the statistical power and a more precise analysis of any associations. Second, this in vitro study could not accurately simulate the intraoral environment, occlusal forces, and wear affecting zirconia bond strength. Methodological changes that could improve the generalizability of the study, such as assessing the outcome of various primers and the prolonged durability of the bonding, could provide detailed insight. This study did not include a control group because of time and resource constraints. Moreover, this study did not utilize more precise methods for surface characterization and surface roughness such as 3D optical profilometry. Furthermore, this study relied on the analysis of shear bond strength, while a more sensitive assessment, such as micro-tensile bond strength, would be favorable for better understanding the adhesive reaction.

**Clinical implications**

This study emphasizes the synergistic effect of combining air abrasion with MDP primer application to improve the bonding of zirconia. The clinical applications of both micromechanical retention and chemical adhesion can be utilized to reduce bond failure. Cautious utilization of air abrasion to avoid the risk of over-treatment of the zirconia surface should be employed to retain the structural integrity of zirconium.

**Recommendations for future research**

The use of sandblasting and MDP primers showed promising outcomes in obtaining an optimal bond strength in relation to zirconia with resin cement. However, future studies should focus on exploring the continued durability of bonding under these surface treatments in the oral environment. Research on the development of standardized treatment protocols for zirconium to achieve optimal adhesion should be prioritized.

## 5. Conclusion

This study showed that the consecutive effect of sandblasting and the 10-MDP primer profoundly enhanced the shear bond strength in zirconia by altering the surface topography micromechanically as well as chemically. When applied in clinical practice, the combined surface treatment protocol can optimize the bonding between resin cements and zirconia, ensuring the durability of zirconia restorations.

## Ethical considerations

The ethical approval for this study was obtained from the Institutional Ethics Committee (Protocol Ref No: 23119, obtained on 15 January, 2024) and was carried out in the Prosthodontics and Crown and Bridge Department of the Manipal College of Dental Sciences, Mangalore.

## Consent to participate

No human participants were involved in this study.

## Data Availability

Figshare: Effect of sandblasting and MDP based primer application on the surface topography and shear bond strength of zirconia: An invitro study, https://doi.org/10.6084/m9.figshare.29222792.v3. ^33^ This project contains the following underlying data:
•
Table - Surface topography of the zirconia discs assessed with Atomic Force Microscopy [Surface topography assessment of the three groups of zirconia discs subjected to various surface modifications assessed with Atomic Force Microscopy] • Elemental composition of the zirconia discs assessed through SEM analysis [Elemental composition of the three groups of zirconia discs subjected to various surface modifications assessed through SEM] Data are available under the terms of the Creative Commons Attribution 4.0 International license (CC-BY 4.0). No extended data beyond those mentioned in this article are available for this study.
