# Supplementary material for: Effect of sandblasting and MDP based primer application on the surface topography and shear bond strength of zirconia: An in vitro study
Source: F1000Res. 2025 Jul 2;14:642. [Version 1] doi: 10.12688/f1000research.166325.1 (PMC12365593; doi:10.12688/f1000research.166325.1)
Supplement: Peer review history

# Reviewer report: Reviewer response for version 1

**DOI:** 10.5256/f1000research.183297.r397592

**Published:** 2025-08-22

**Authors:** Awiruth Klaisiri

**Affiliations:** 1 Thammasat University, Pathum Thani, Thailand

While the study addresses a relevant topic and is conducted with acceptable methodology, it appears to offer limited novel contributions to the existing body of knowledge. The findings largely confirm results from prior research, and there is no clear indication of how this study advances the current understanding of the subject matter. Furthermore, the discussion does not sufficiently highlight any unique insights or theoretical implications that differentiate it from previously published work. The authors are encouraged to clarify the originality of their work and explicitly state how their findings contribute to the field, which would strengthen the manuscript’s overall impact.

Is the work clearly and accurately presented and does it cite the current literature?

Partly

If applicable, is the statistical analysis and its interpretation appropriate?

Yes

Are all the source data underlying the results available to ensure full reproducibility?

Yes

Is the study design appropriate and is the work technically sound?

Partly

Are the conclusions drawn adequately supported by the results?

Partly

Are sufficient details of methods and analysis provided to allow replication by others?

Partly

Reviewer Expertise:

Dental material sciences

I confirm that I have read this submission and believe that I have an appropriate level of expertise to state that I do not consider it to be of an acceptable scientific standard, for reasons outlined above.

# Reviewer report: Reviewer response for version 1

**DOI:** 10.5256/f1000research.183297.r397584

**Published:** 2025-08-19

**Authors:** Aditi Nanda

**Affiliations:** 1 All India Institute of Medical Sciences, New Delhi, India

Here's a 150-word review of the article, including its positives and negatives:

 This in vitro study effectively investigates the synergistic impact of sandblasting and MDP primer application on zirconia's surface topography and shear bond strength. The authors clearly demonstrate that the combined treatment significantly enhances both surface roughness and shear bond strength, with Group C (combined treatment) showing the highest bond strength (16.05 ± 8.91 MPa). The analysis of failure modes further supports the superior bonding with a higher proportion of cohesive and mixed failures in Group C. This study provides valuable evidence supporting a combined surface treatment approach for improved zirconia restoration durability in clinical practice.

 Positives **:**

- Clear objective and methodology **:** The study design is well-defined, and the experimental groups are distinct, allowing for clear comparisons.
- Comprehensive analysis **:** The use of AFM, SEM, EDS, and shear bond strength tests provides a multi-faceted evaluation of the surface modifications and their effects.
- Clinically relevant findings **:** The results directly inform better bonding protocols for zirconia restorations, which is highly practical for dentistry.
- Strong evidence for combined treatment: The study robustly supports the superiority of combined sandblasting and MDP primer application.

Negatives:

- Small sample size: A sample size of 10 per group is relatively small, which the authors acknowledge may have affected statistical significance for failure modes.
- In vitro limitations **:** The study cannot fully replicate the complex intraoral environment, including factors like occlusal forces, thermal changes, and saliva contamination.
- Lack of control group: The absence of an untreated control group makes it harder to definitively quantify the individual impact of each treatment against a baseline.
- Limited surface characterization methods: The authors mention that more precise methods for surface characterization, like 3D optical profilometry, were not used.

Is the work clearly and accurately presented and does it cite the current literature?

Yes

If applicable, is the statistical analysis and its interpretation appropriate?

Yes

Are all the source data underlying the results available to ensure full reproducibility?

Yes

Is the study design appropriate and is the work technically sound?

Yes

Are the conclusions drawn adequately supported by the results?

Yes

Are sufficient details of methods and analysis provided to allow replication by others?

Yes

Reviewer Expertise:

Prosthodontics and dental materials

I confirm that I have read this submission and believe that I have an appropriate level of expertise to confirm that it is of an acceptable scientific standard.

# Reviewer report: Reviewer response for version 1

**DOI:** 10.5256/f1000research.183297.r397588

**Published:** 2025-08-14

**Authors:** Gopi Chander

**Affiliations:** 1 Department of Prosthodontics, SRM Dental College, Chennai, Tamil Nadu, India

The study addresses the ongoing challenge of bonding to zirconia, a material of growing importance in prosthodontics due to its favorable mechanical and esthetic properties.

 The focus on MDP primers and sandblasting reflects current trends and debates in clinical adhesive protocols for zirconia.

 The manuscript has a clear aim: to evaluate the synergistic effect of sandblasting and MDP-based primers on zirconia.

 It appropriately combines mechanical (sandblasting) and chemical (MDP primer) treatments for comprehensive insight.

 The study uses a controlled in vitro design with clearly defined experimental groups (MDP only, sandblasting only, and combination treatment).

 High-quality, **complementary surface analysis tools** were used:

 A universal testing machine and a customized setup ensure standardized shear bond strength measurement.

 Bond strength values are supported by statistical analysis, and failure modes are categorized and compared.

 Use of **ANOVA**, **Tukey's post-hoc**, and **chi-square tests** supports the reliability of findings. Use of p < 0.05 for statistical significance is consistent with standard practice.

 The manuscript is logically structured: background, methods, results, and conclusion are well-separated.

 The study contributes to standardizing a dual-treatment protocol for zirconia surface conditioning.

 It attempts to bridge inconsistencies in literature by keeping abrasive parameters (particle size, pressure, duration) constant.

 Findings offer immediate relevance for clinical practice—suggesting that combined MDP and sandblasting treatments yield superior bond strength. It contributes evidence for practitioners selecting surface treatment protocols for zirconia restorations.

Is the work clearly and accurately presented and does it cite the current literature?

Yes

If applicable, is the statistical analysis and its interpretation appropriate?

I cannot comment. A qualified statistician is required.

Are all the source data underlying the results available to ensure full reproducibility?

Yes

Is the study design appropriate and is the work technically sound?

Yes

Are the conclusions drawn adequately supported by the results?

Yes

Are sufficient details of methods and analysis provided to allow replication by others?

Yes

Reviewer Expertise:

dental materials, prosthodontics

I confirm that I have read this submission and believe that I have an appropriate level of expertise to confirm that it is of an acceptable scientific standard.
